# Systematic review and meta-analysis of second-generation antidepressants for the treatment of older adults with depression: questionable benefit and considerations for frailty

**DOI:** 10.1186/s12877-019-1327-4

**Published:** 2019-11-12

**Authors:** Laurie Mallery, Tanya MacLeod, Michael Allen, Pamela McLean-Veysey, Natasha Rodney-Cail, Evan Bezanson, Brian Steeves, Constance LeBlanc, Paige Moorhouse

**Affiliations:** 1 Division of Geriatric Medicine, Dalhousie University, Halifax, Nova Scotia Canada; 2 Continuing Professional Development, Dalhousie University, Halifax, Nova Scotia Canada; 3 Drug Evaluation Unit, Nova Scotia Health Authority, Halifax, Nova Scotia Canada; 4 Sobeys National Pharmacy Group, Halifax, Nova Scotia Canada; 5 RK MacDonald Nursing Home, Halifax, Nova Scotia Canada

**Keywords:** Antidepressants, Older adults, Frail older adults, Systematic review, Meta-analysis, Depression

## Abstract

**Background:**

Frail older adults are commonly prescribed antidepressants. Yet, there is little evidence to determine the efficacy and safety of antidepressants to treat depression with concomitant frailty. To better understand this issue, we examined the efficacy and safety of second-generation antidepressants for the treatment of older adults with depression and then considered implications for frailty.

**Methods:**

Due to the absence of therapeutic studies of frail older adults with depression, we conducted a systematic review and meta-analysis of double-blind, randomized controlled trials that compared antidepressants versus placebo for adults with depression, age 65 years or older. We searched PubMed/MEDLINE, Cochrane Library, reference lists from meta-analyses/studies, hand searches of publication lists, and related articles on PubMed. Outcomes included rates of response, remission, and adverse events. After evaluating the data, we applied a frailty-informed framework to consider how the evidence could be applied to frailty.

**Results:**

Nine trials were included in the meta-analysis (*n* = 2704). Subjects had moderate to severe depression. For older adults with depression, there was no statistically significant difference in response or remission to second-generation antidepressants compared to placebo. Response occurred in 45.3% of subjects receiving an antidepressant compared to 40.5% receiving placebo (RR 1.15, 95% CI: 0.96 – 1.37, *p* = 0.12, I2 = 71%). Remission occurred in 33.1% with antidepressant versus 31.3% with placebo (RR 1.10, 95% CI: 0.92 – 1.31, *p* = 0.30, I2 = 56%) (Figure 2 and 3). There were more withdrawals due to adverse events with antidepressants, 13% versus 5.8% (RR 2.30, 95% CI: 1.45–3.63; *p* < 0.001; I^2^ = 61%; NNH 14, 95% CI:10–28).

**Implications for frailty:**

Subjects in the meta-analysis did not have obvious characteristics of frailty. Using framework questions to consider the implications of frailty, we hypothesize that, like older adults, frail individuals with depression may not respond to antidepressants. Further, observational studies suggest that those who are frail may be less responsive to antidepressants compared to the non-frail. Given the vulnerability of frailty, adverse events may be more burdensome.

**Conclusions:**

Second-generation antidepressants have uncertain benefit for older adults with depression and cause more adverse events compared to placebo. Until further research clarifies benefit, careful consideration of antidepressant prescribing with frailty is warranted.

## Background

Frailty is associated with an increased risk of being diagnosed with depression [1–3]. Further, in long-term care settings, where many frail older adults reside, antidepressants are amongst the most commonly prescribed medications [4, 5]. Thus, it is important to consider how frail patients with depression respond to treatment. Nevertheless, there is little written about the efficacy and safety of antidepressants for the treatment of frail older adults with depression. To address this knowledge gap, we employed standardized methodology to review and critique the evidence for older adults, followed by theoretical consideration of its applicability to frailty.

## Methods

Our original goal was to assess the efficacy and safety of antidepressants for the treatment of frail older adults with depression but without dementia. However, we found no double-blind, randomized, controlled trials (DBRCTs) that specifically evaluated the use of antidepressants in the frail population. Thus, we evaluated the efficacy and safety of antidepressants for older adults with depression. Due to the convention that a person aged 65 years or older is often referred to as ‘elderly’, [6] we pre-specified that age group for inclusion, as is commonly done in evidence reviews of older adults [7]. We subsequently considered how results obtained for the older population might apply to frailty.

We performed a systematic review and meta-analysis in accordance with PRISMA (Preferred Reporting Items for Systematic reviews and Meta-Analyses) guidelines [8].

Funding was provided by a Knowledge Synthesis grant (FRA 2015-A-04) from the Canadian Frailty Network and the Nova Scotia Department of Health and Wellness, as well as in-kind contributions from Dalhousie University Continuing Professional Development, the Canadian Agency for Drugs and Technologies in Health (CADTH), and Sobeys National Pharmacy Group. The funders had no role in study design, data collection and analysis, decision to publish, or preparation of the manuscript.

### Inclusion criteria

Due to the significant placebo response of antidepressants in clinical trials, we considered only DBRCTs of adults 65 years or older. We included studies of subjects who had been diagnosed with depression according to Diagnostic and Statistical Manual of Mental Disorders (DSM) criteria or achieving a pre-specified rating on a validated depression scale. We focused on second-generation antidepressants, as these are most commonly prescribed for older adults, and included trials of SSRIs, serotonin-norepinephrine reuptake inhibitors (SNRIs), and noradrenergic and specific serotonergic antidepressants (NaSSAs).

We reviewed publications of DBRCTs, meta-analyses, and systematic reviews that provided response and remission data. As the response to antidepressants with dementia may differ from those without dementia, [9] we aimed to exclude studies that enrolled individuals with dementia. Several trials incompletely reported on the number of subjects with dementia. To address this potential shortcoming, we conducted a sensitivity analysis of the trials that explicitly excluded older adults with dementia. We limited studies to English language publications.

### Search strategy

We searched PubMed/MEDLINE, Cochrane Library, and reference lists from meta-analyses/studies, hand searches of publication lists, and related articles featured on PubMed. We used the search terms (antidepressants AND depression) with specified limits of meta-analysis; systematic review; randomized controlled trial; humans; English language; and aged: 65+ years that were published between January 1, 1996 and March 1, 2016. Studies were downloaded and screened using Covidence online software [10]. Two investigators (LM, TM) independently conducted study selection. When there were divergent views, the reviewers presented their opinions to the team for consensus decision.

### Data collection process

Three members of the review team extracted and/or verified data from each study (MA, EB, TM). Data was double checked by LM and discrepancies resolved through discussion. We used a Microsoft Excel™ extraction sheet to catalog information including: study purpose; design/year/setting; drug/comparator mean dose; participant numbers; trial duration; study withdrawals; inclusion/exclusion criteria; assessment procedures/rating scales; baseline demographics; statistical adjustments; study results for primary/secondary outcomes; adverse events; withdrawals; mortality; subgroup analyses; reviewer conclusions; and risk of bias criteria.

### Risk of bias assessment and study quality

Two authors (MA, EB) assessed study quality and risk of bias in individual studies using GRADE criteria [11] and Cochrane risk of bias criteria [12]. There was extensive discussion on study design and interpretation of results by all authors in regularly scheduled meetings.

### Synthesis of results

We used RevMan™ 5.3 to calculate pooled effect sizes and 95% confidence intervals using a random-effects model. Heterogeneity was assessed using the I^2^ statistic.

We used an alpha level for statistical significance of *p* ≤ 0.05 and calculated number needed to treat/number needed to harm (NNT/NNH) by applying the pooled estimate of relative risk (RR) to the pooled event rate in the placebo group.

### Outcome measures

Primary outcomes were response and remission of depression. We defined response as a decrease of 50% or greater in depression scores or a Clinical Global Impression-Improvement (CGI-I) Scale score ≤ 2 at the final visit, which rates improvement in symptoms as “better” or “much better.” Remission was determined using the definitions in the included studies based on the following scores: Hamilton Depression Rating Scale (HDRS)-24 ≤ 10, HDRS-17 ≤ 7, or Montgomery-Åsberg Depression Rating Scale (MADRS) ≤ 11 or ≤ 12. If more than one scale was reported, we gave priority to HDRS, as was done in an earlier meta-analysis [13].

We pooled data for specific adverse events and only analyzed adverse events for which there were sufficient data to conduct a meta-analysis of results, which included withdrawals due to adverse events, dry mouth, dizziness, constipation, headache, diarrhea, nausea, somnolence, insomnia, and fatigue.

Summary results are presented using relative and absolute risk statistics and 95% confidence intervals. Concluding statements were derived from team consensus based on meta-analysis findings, assessment of study quality, and application to frailty based on the guiding questions of the framework.

## Results

### Search results and risk of bias assessment

**Fig. 1:**
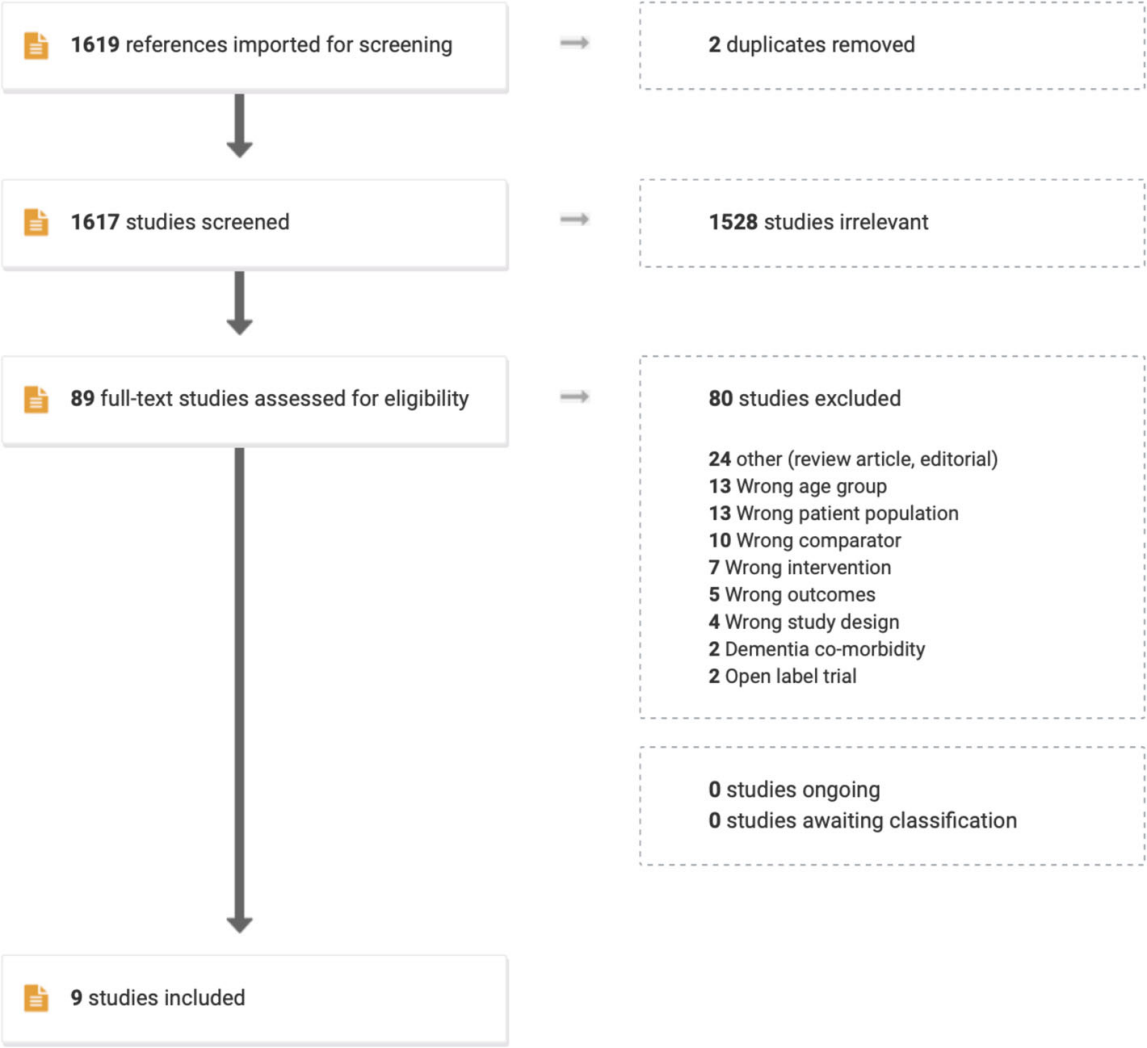
Flow Diagram of Trial Identification and Selection Process

Initially, 1619 abstracts were identified. Of these, 1530 were excluded as they were either duplicates, reports that addressed other topics/populations, or not randomized controlled trials of second-generation antidepressants. Abstracts or published articles for the remaining 89 clinical trials were obtained and thoroughly reviewed. Of 89 potential trials, 80 were excluded for reasons listed in Fig. 1.

Nine DBRCTs met our inclusion criteria (Table 1) [14–22].

**Table 1:** Studies of subjects ≥65 years included in the meta-analysis

Author Year	Drug & Dose	Time (weeks)	N	Mean Age (years)	Quality^a^	Scale	Primary outcome	Statistical benefit
Evans 1997 [14]^b^	FLU 20 mg	8	62	82	Low^d^	HDRS-17	Response	No
Fraguas 2009 [15]^bc^	CIT 20-40 mg	8	37	74	Low	HDRS-17	Response	No
Hewett 2010 [16]	BUP 105–300 mg	10	418	71	Low^d^	MADRS	Change from baseline	Inconsistent^e^
Kasper 2005 [17]	ESC 10 mg FLU 20 mg	8	517	75	Moderate^d^	MADRS	Change from baseline	No
Katona 2012 [18]	DUL 60 mg	8	452	71	High^d^	HDRS-24	Change from baseline	Yes
Raskin 2008 [19] ^b^	DUL 60 mg	8	311	73	Low^d^	HDRS-17	Response/ remission	Yes
Robinson 2014 [20]^b^	DUL 60 mg	12 + 24 extension	370	73	Low^-d^	HDRS-17 Maier subscale	Change from baseline	No
Roose 2004 [21]	CIT 10–20 mg	8	174	80	High^d^	HDRS	Response/ remission	No
Schatzberg 2006 [22]^b^	FLU 40–60 mg VEN 150–225 mg	8	300	71	High^d^	HDRS-21	Response/ remission	No

^a^ Quality based on Cochrane risk of bias criteria; ^b^ Enrolled subjects with dementia/did not explicitly exclude dementia subjects; ^c^ Subjects had heart failure; ^d^ Trial had industry funding/industry employees as authors; ^e^ The pre-specified ANCOVA analysis was not statistically significant (*p* = 0.09). Post-hoc rank-based ANCOVA analysis was statistically significant (*p* = 0.03). Response was statistically significant (*p* = 0.01), but remission was not statistically significant (*p* = 0.17)

*FLU* Fluoxetine, *CIT* Citalopram, *BUP* Buspirone, *ESC* Escitalopram, *DUL* Duloxetine, *VEN* Venlafaxine, *HDRS* Hamilton Depression Rating Scale, *MADRS* Montgomery-Åsberg Depression Rating Scale

Blinding and allocation concealment were often inadequately described. Five of the nine studies used intention-to-treat analysis (ITT), [16–18, 21, 22] while the other four stated they used ITT analysis that was not borne out by close analysis of the number of subjects in the results [14, 15, 19, 20]. Seven studies had over 100 subjects [16–22] and two studies had fewer than 65 subjects [14, 15]. No studies adjusted *p*-values for multiple comparisons.

All studies, except one small study, [15] were funded by industry. Using the GRADE criteria for study quality, [11] we judged five studies to be of low quality, [14–16, 19, 20] one of moderate quality, [17] and three of high quality [18, 21, 22] (Table 1).

### Study outcomes

The mean age of participants ranged from 71 to 82 years (Table 1) and only one study was limited to subjects who were 75 years of age or older [21]. Subjects were mostly community-dwelling, with one study including inpatients on a geriatric medical unit [14]. Based on mean depression scores, the study subjects had moderate to severe depression. In three studies, the majority of participants had recurrent episodes of depression [16, 18, 19]. The other studies did not specify the number of subjects with previous depression [14, 15, 17, 20–22].

**Fig. 2:**
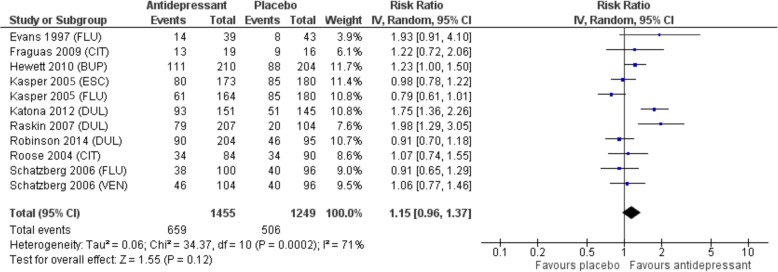
Forest plot demonstrating no overall difference in response for second generation antidepressants versus placebo

**Fig. 3:**
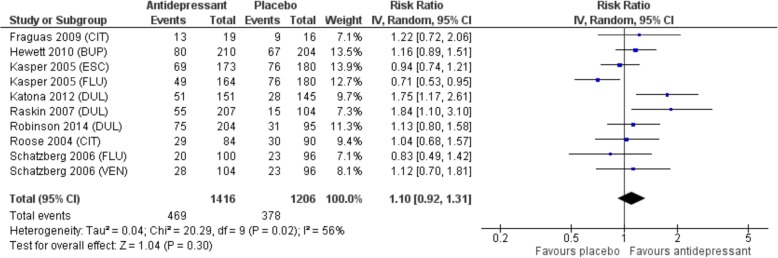
Forest plot demonstrating no overall difference in remission for second generation antidepressants versus placebo

There was no statistically significant difference in response or remission to second-generation antidepressants compared to placebo. Response occurred in 45.3% of subjects receiving an antidepressant compared to 40.5% receiving placebo (RR 1.15, 95% CI: 0.96–1.37, *p* = 0.12, I^2^ = 71%). Remission occurred in 33.1% of those given an antidepressant versus 31.3% with placebo (RR 1.10, 95% CI: 0.92–1.31, *p* = 0.30, I^2^ = 56%) (Fig. 2 and 3).

Since older adults with depression and concomitant dementia might have a different response to antidepressants compared to those without dementia, [9] we examined studies that explicitly excluded subjects with dementia, which was the case in four of nine studies [16–18, 21]. We conducted a sensitivity analysis of these trials and found no statistically significant response (RR 1.12, 95% CI: 0.86–1.46, *p* = 0.38, I^2^ = 81%) or remission (RR 1.05, 95% CI: 0.81–1.37, *p* = 0.72, I^2^ = 72%) with antidepressants compared to placebo.

Reporting of adverse events was inconsistent. Nevertheless, we combined frequently reported events in the meta-analysis. The most common adverse event was nausea, with a rate of 14% for second-generation antidepressants and 6.8% for placebo (RR 2.26, 95% CI: 1.42–3.60; *p* = 0.001; I^2^ = 63%; NNH 11, 95% CI:7–30). Seven studies (*N* = 2444 subjects) recorded withdrawals due to adverse events, which occurred more frequently with second-generation antidepressants compared to placebo, 13% versus 5.8% (RR 2.30, 95% CI: 1.45–3.63; *p* < 0.001; I^2^ = 61%; NNH 14, 95% CI:10–28). Other adverse events occurring more frequently in the antidepressant group were fatigue (9.4% vs 4.6%, *p* = 0.002); constipation (9.5% vs 4.2%, p = 0.002); and dizziness (7.3% vs 5.6%, *p* = 0.008).

There was no statistically significant difference between second-generation antidepressants and placebo for headache, diarrhea, dry mouth or insomnia. Only one study specifically reported on fall rates [20] and one on hyponatremia [17], with neither study showing statistically significant differences between groups. No studies reported on QTc prolongation or serious adverse events, such as death or cardiovascular events.

## Discussion

### Consideration of frailty

After evaluating the evidence for older adults, we considered how the evidence could apply to frailty.

#### What is frailty?

Frailty describes the loss of physiologic reserve that results in vulnerability to adverse events [2, 23–25]. Frailty can be identified in several ways, such as with the Clinical Frailty Scale [26] or FACT tool, [27] both of which define frailty as the lifelong accumulation of health issues that result in declining function, mobility, or cognition. Although there is heterogeneity among frail patients, frailty is generally progressive and robustly correlates with increased mortality and dependency [23]. In contrast, successful aging is associated with illness avoidance, high physical and cognitive function, and active engagement in life [28].

#### Frailty and polypharmacy

There is growing awareness about the importance of reducing unnecessary medications for older adults but little consensus about the best way to approach medication appropriateness [29]. One popular strategy is to use tools like the Beers [30] or STOPP criteria, [31] which identify potentially inappropriate medications. While these tools have made important contributions to clinical care, they have several limitations. First, adverse drug events are often related to non-list medications. In one study, [32] Beers criteria medications caused low numbers of emergency department visits compared to non-list medications, such as warfarin, insulin, and digoxin. Also, lists may inappropriately discourage the use of medications to meet individual circumstances, such as using antipsychotic medications to palliate severe behavioral symptoms in advanced dementia. Third, despite admonitions to avoid certain medications, health professionals still endeavor to treat symptoms and may prescribe similarly risky non-list medications; for example, treating insomnia with trazodone to avoid using a benzodiazepine. Finally, the Beers and STOPP criteria do not address the degree to which individuals with advanced frailty should adhere to standard clinical guidelines for common medical conditions, a practice that can result in large numbers of prescribed medications and, thus, become a significant driver of polypharmacy [33]. Therefore, one way to improve prescribing appropriateness is to develop frailty-specific recommendations— an approach we have adopted for the treatment of hypertension, diabetes, and prevention of cardiovascular disease with statins [27, 34, 35].

#### Frailty and clinical trial evidence

Due to age and/or comorbidity-related exclusion criteria, almost all drug trials exclude individuals who are frail [36]. As such, there is rarely specific data to inform the treatment of frail older adults, who may not respond in the same way as healthier populations. Frail individuals are at increased risk of medication interactions and medication-related adverse events, [37] which alters the risk-to-benefit ratio. Frailty also presents competing risks for morbidity and mortality, whereby the improvement of one health issue may be camouflaged by decline in another [38]. Finally, characteristics of frailty—such as shortened life expectancy, cognitive impairment, and functional decline—may minimize the impact of intended therapeutic benefit.

#### Rationale for a focus on antidepressants

Frail older adults have high rates of depression. Two reviews [1, 3] show that those who are frail are at increased risk of having depression, even after adjusting for potential confounders. In a landmark study, Fried [2] reported that 31% of frail older adults had a “suggestive diagnosis of depression” compared to only 3% of non-frail elders. As well, medical conditions that are commonly associated with frailty have high rates of depression—approximately 31% with stroke, [39] 22% with heart failure, [40] 23% with Parkinson’s disease, [41] and 32% with mild cognitive impairment [42].

In the case of late-life depression, the prevailing opinion is that older adults are underdiagnosed and undertreated for depression [43]. Yet, among frail older adults, the opposite may be true. In Canadian long-term care, where the majority of older adults are typically severely frail, roughly 44% have a diagnosis and/or symptoms of depression and nearly 60% use an antidepressant, although possibly for diverse indications [4]. In U.S. nursing homes, 54% of residents were diagnosed with depression—33% at admission and a further 21% during the first year [5]. The authors of that study conclude that “the high antidepressant use in nursing homes may reflect a spiraling trend of over-diagnosing [and treating] depression.”

#### A frailty-informed framework to evaluate evidence

As there is insufficient evidence specific to the frail, we considered how results from studies of older adults could be relevant to those who are frail using a frailty-informed framework that poses five questions [35]. These questions focus on features of frailty that could affect the applicability of evidence, such as: 1) characteristics of the trial population; 2) outcomes; 3) timeline for benefit; 4) harms; and 5) other relevant evidence. This inquiry, used in earlier reviews, [27, 34, 35] is conducted by an interdisciplinary team of evidence appraisal specialists, pharmacists, family physicians, and geriatricians.

Application of the framework questions resulted in the following analysis:

1. How does the study population compare with those who are frail?

The subjects in our meta-analysis were generally younger and healthier than those who are frail. All studies excluded subjects with unstable medical conditions and other psychiatric syndromes (Table 2). However, one study enrolled subjects with stable heart failure [15] and in another study, most subjects had one or more health problems [14]. As individuals with heart failure and medical illness have a high prevalence of frailty, [44] these two studies may more closely represent the frail population. Both showed that antidepressants were no more effective than placebo.

**Table 2:** Inclusion/exclusion criteria and characteristics of participants from included studies

Inclusion and study characteristics	Exclusion
Diagnosis of Major Depressive Disorder according to DSM and/or achieving a pre-specified rating on a depression scale	Treatment-resistant depression
Moderate to severe depression based on standard clinical measures of depression (e.g., HDRS, MARS)	Complex depressive disorders, such as bipolar disorder, depression with psychotic features, dysthymic disorder, neurotic depression, or minor depression
Age ≥ 65 years (only one study included patients ≥75 years old [21])	Comorbid alcohol disorders or substance abuse disorders
• Subject were mostly outpatients, although this was not always specified • One study enrolled only inpatients admitted under the care of a geriatrician or family physician [14]	Unstable medical conditions, although two studies allowed inclusion of patients with concomitant medical conditions. Fraguas studied patients with stable heart failure [15] and Evans included patients with other medical conditions, including dementia [14].
Not at risk of suicide	Four of nine studies specifically excluded subjects with dementia [16–18, 21]. The other five studies either did not explicitly state the exclusion of subjects with dementia or enrolled subjects with mild stage dementia [14, 15, 19, 20, 22].

*DSM* Diagnostic and Statistical Manual of Mental Disorders, *HDRS* Hamilton Depression

Rating Scale; *MARS* Medical Administration Record Sheet

Similar to this meta-analysis, a systematic review of randomized controlled trials of antidepressants for older adults with late-life depression [45] found that “geriatric characteristics” were rarely taken into account or considered as co-variables and that the oldest adults were underrepresented in these clinical trials. The authors, thus, questioned whether evidence for treating major depression had sufficient external validity for the heterogenic population of older adults.

2. Are study outcomes relevant to those who are frail?

Outcomes that are relevant for healthier adults may not be relevant with frailty. Therefore, we consider how an outcome might relate to overall health when individuals are frail.

In our meta-analysis, primary and secondary outcomes were response and remission based on Depression Rating Scales. However, it is not clear whether these rating scales can differentiate symptoms of depression from characteristics of frailty and whether measured change represents meaningful benefit. In particular, DSM-5 criteria for major depression and depressive symptoms overlap with common manifestations of both frailty and chronic health conditions (Table 3). When individuals are frail, conditions such as functional disability, cognitive decline, impaired mobility, and/or physical symptoms may give rise to features commonly attributed to depression, such as fatigue, limited activity, decreased interest, trouble sleeping, feelings of sadness, and/or thoughts of death. Medications, such as those used to treat pain, may impair concentration. In addition, old age commonly brings challenging circumstances, such as the loss of a spouse or financial insecurity, which can lead to despondency. Indeed, Lohman [46] postulated that the strong correlation between frailty and depression could be related to the criteria used in their measurement and concluded that that available measures of frailty and depression are either poor at discriminating between the two constructs or identify the same underlying condition.

**Table 3:** Overlapping symptoms of depression and frailty

Symptom	Depression (DSM-5)^a^	Possible in frailty
Depressed mood/irritability	Y	Y
Loss of interest	Y	Y
Weight change	Y	Y
Reduced activity	Y	Y
Fatigue/loss of energy	Y	Y
Change in sleep	Y	Y
Decreased concentration	Y	Y
Guilt and feeling worthless	Y	Maybe
Suicidality	Y	Thoughts of dying

^a^Five or more of the symptoms are present during the same two-week time frame and represent a change from previous functioning. At least one of the symptoms is either (1) depressed mood or (2) loss of interest or pleasure. Additionally, symptoms cause clinically significant distress or impaired function and are not attributable to the physiological effects of a substance or to another medical condition

*DSM* Diagnostic and Statistical Manual of Mental Disorders

3. Is the timeframe relevant for those who are frail?

Given the shortened life expectancy associated with frailty and the expected progression of frailty over time, treatment benefits that accrue over many years may not be applicable to the frail, while studies of short duration may underestimate risk.

In this meta-analysis, study duration ranged from 8 to 12 weeks, a reasonable timeframe to achieve benefit. However, none of the studies addressed the sustainability of response nor the likelihood of developing adverse effects as frailty increases over time. In one 12-week study that had a 12-week extension, [20] falls were more frequent with duloxetine compared to placebo over 24-weeks that included the acute plus continuation phase (24% vs 14%, *p* = 0.04) but not in the first 12 weeks (16% vs 10%, *p* = 0.15).

4. Have potential harms been sufficiently considered?

Since the frail are vulnerable, medication adverse effects may impact their quality of life and health status to a greater extent compared to healthier adults. Thus, both potential risks and benefits related to treatment need to be equally considered in the context of frailty.

The harms reported in this meta-analysis appear to be minor. However, nausea, fatigue, constipation, and dizziness were more frequent with antidepressants compared to placebo, which may be burdensome for frail older adults who are less able to tolerate perturbations in health due to decreased reserve. Notably, rates of withdrawal due to adverse events in subjects receiving antidepressants was twice the rate for those receiving placebo. In addition, frail older adults with multiple co-morbidities are at risk of polypharmacy. As the number of medications increase, so too does the potential for adverse medication events related to antidepressants.

5. Is there further evidence pertaining to frail populations?

Although frail patients may not be specifically enrolled in randomized controlled trials, other categories of evidence can shed light on frailty response. Here, we consider two sources: (1) examination of medical conditions that are significantly associated with frailty; and (2) observational studies.

We deliberated on two medical conditions that are associated with high rates of frailty—heart failure and Parkinson’s disease.

With heart failure, a systematic review and meta-analysis showed that the overall estimated prevalence of frailty was 44.5% (95% CI: 36.2–52.8%, z = 10.54, *p* < 0.001) [44]. Similarly, a substantial number of individuals with Parkinson’s disease would be considered frail, as this illness typically affects mobility.

Two DBRCTs of adults with heart failure found that antidepressants did not improve depression compared to placebo. The Sertraline Against Depression and Heart Disease in Chronic Heart Failure (SADHART-CHF) [47] included 469 subjects with a mean age of 62. At 12 weeks, there was no significant difference in depression scores for sertraline compared to placebo. Similarly, the Mortality, Morbidity, and Mood in Depressed Heart Failure Patients (MOOD-HF) trial, [48] which included 372 adults with a mean age of 62 years, showed that there was no significant improvement in depression with escitalopram compared to placebo.

We examined five meta-analyses of antidepressants for depression in Parkinson’s disease [49–53]. Three of the meta-analyses found insufficient evidence to support the use of antidepressants for the treatment of depression with Parkinson’s disease [49–51]. Two meta-analyses reached a different conclusion and found that antidepressants significantly improved depression with Parkinson’s disease, [52, 53] although one of these meta-analyses included trials without a placebo arm [53] and the other included a study that enrolled subjects without depression [52].

In our meta-analysis, the two studies that enrolled subjects with depression and concomitant medical conditions, likewise, showed no statistically significant benefit from antidepressants [14, 15].

Although randomized trials are the best way to determine medication efficacy, observational studies may suggest potential associations. Three observational studies support the hypothesis that those who are frail may be less responsive to antidepressants compared to the non-frail. A multi-site naturalistic prospective cohort study from the Netherlands of 378 subjects over age 60 [54] found that depression with comorbid frailty was less likely to resolve compared to depression unaccompanied by frailty. In that study, frail patients achieved 2-year remission significantly less often than their robust counterparts (55.4% versus 30.6%, χ^2^ = 8.3, df = 2, *P* = .016). Analogously, in a longitudinal study [55] of 189 persons with depressed mood, remission was less likely with higher levels of physical frailty (hazard rate = 0.72, 95% confidence interval 0.58–0.91, *P* = .005). Finally, using data from the Nordic Research on Ageing (NORA), [56] Brown found that the combination of late-life depression and frailty was associated with increased likelihood of poor outcomes. In that study, depressed older women with frailty had higher death rates compared to those who were frail but not depressed.

### Consideration of results

In our meta-analysis of older adults with depression, there was no statistically significant response or remission for second-generation antidepressants compared to placebo. Our results are similar to a meta-analysis by Tedeschini, [13] who also reported no significant treatment effect with antidepressants versus placebo in those over 65 years of age, although the authors cautioned that this finding was limited by a small number of trials (*n* = 5). In contrast, when the Tedeschini meta-analysis employed an age threshold of > 55 years, a statistically significant benefit for antidepressants was found, which raised the possibility that there may be less response to antidepressants in later life, according to the authors. With the addition of four trials, [14, 15, 18, 20] our review builds on this earlier meta-analysis.

Our review highlights the absence of clinical trials for frail older adults with depression. Subjects enrolled in our meta-analysis of older adults did not have obvious characteristics of frailty. Yet, treatment with antidepressants appears to be common in the frail population, as evidenced by the high rate of antidepressant use among long-term care residents, where there is a high prevalence of frailty [4, 5].

So, what can be theorized about the expected response to antidepressants for frail older adults with depression? If non-frail older adults with depression do not exhibit response or remission to antidepressants, frail adults would, likewise, not be expected to respond. In fact, preliminary data from longitudinal cohort studies imply that frail individuals respond less favorably to antidepressants compared to older adults who are not frail, which could be related to several factors. First, although major depression can co-exist with frailty, there is overlap between symptoms of depression and the characteristics of frailty, as described above [46, 57]. An erroneous diagnosis of depression with frailty may result in an antidepressant prescription for the wrong indication. Another concern is that depression scales used in trials may not be clinically relevant to those who are frail as they measure characteristics common to frailty. Finally, in clinical settings, it may be difficult to differentiate medication from placebo response, as roughly 40% of subjects in the meta-analysis demonstrated response to placebo.

Frailty is associated with increased potential for adverse events [23–25]. As such, the possibility of harm from antidepressants warrants careful consideration, as they may be less well tolerated in frailty compared to those without frailty. In this meta-analysis of older adults without frailty, 13% of subjects in the treatment arm withdrew because of adverse events compared to 5.8% with placebo (NNH = 14), with nausea reported most frequently (NNH = 11). No study reported the incidence of Q-Tc prolongation and only one study reported on hyponatremia, [17] both of which are known adverse effects of antidepressants [58]. The trials reported only short-term outcomes, which raises the possibility that adverse effects may have been underestimated [59]. In a retrospective cohort study that followed 60,746 older adults with depression for a mean duration of 5.0 years (SD 3.3) [60], adverse events were more frequent with all classes of antidepressants compared to no antidepressant, including mortality, falls and fractures.

### Limitations

This review has several limitations. The focus on adults above age 65 could be viewed as an arbitrary cut-off. However, 65 years is generally accepted as a transition into older age, [6] and the mean age of subjects included in the meta-analysis ranged from 71 to 82 years. Next, risks associated with under-treating high-risk depression were not fully considered, as most studies excluded those at risk of suicide. Third, the high degree of heterogeneity could indicate variable response to antidepressants based on duration of symptoms, severity, or number of recurrences. Fourth, several studies included older adults with dementia, which could negate the results for those without dementia, a concept supported by a Cochrane review, which concluded that available evidence “does not provide strong support for the efficacy of antidepressants for treating depression in dementia.” [9] However, in our sensitivity analysis of trials that excluded older adults with dementia, there was no statistically significant response or remission for antidepressants compared to placebo. Next, this meta-analysis compared anti-depressants to placebo. A network meta-analysis might find a difference between specific agents, which have not been tested in head to head clinical trials. Finally, the exploration of how frail older adults might respond to antidepressants is theoretical and not based on study data. Thus, it is not possible to reach definitive conclusions. The lack of evidence for the frail elderly is a call to action to include frail older adults in clinical trials so that evidence-based practice guidelines can be developed for this population.

## Conclusions

### Recommendations

Considering the possibility that a frail patient may have limited response to antidepressants and is more at risk for adverse events, we recommend the following:

- Use antidepressants judiciously, with awareness of the limited evidence for efficacy in adults age ≥ 65 years and, by association, older adults with frailty.
- Attentively consider the potential for adverse effects with frailty.
- Reflect on patient circumstances and carefully assess whether changes in mood could be situational and/or related to frailty; make a concerted effort to provide the kind of support that can benefit frail older adults, such as home and situational supports.
- Consider that, due to varied populations and heterogeneous response, antidepressants may be beneficial for some older adults who are frail.
- If antidepressants are trialed, adequate dose and treatment time should be assured before judging efficacy. On the other hand, continuing antidepressants when they are not effective contributes to polypharmacy. Therefore, when treatment is initiated, attentively assess treatment response with the aim of stopping medications when no benefit is observed. When making treatment decisions, consider the high placebo response.
- Routinely re-evaluate whether antidepressants should be continued when they are used for prolonged periods of time.
- Reconsider guidelines that recommend screening for depression in frail populations (such as in nursing homes), as this approach likely contributes to the over-diagnosis of depression and polypharmacy.

To conclude, many authors conjecture that “late-life depression remains underdiagnosed and inadequately treated.” [43, 61, 62] In contrast, based on the evidence reviewed and its hypothesized applicability to frailty, we are concerned that frail older adults might be over treated with second-generation antidepressants with little to no benefit.

Our findings contribute to the growing discourse of uncertain clinical benefit from antidepressants and high risk of bias in antidepressant trials [63, 64]. Some experts may not agree with our interpretation of the evidence. They may focus more on the potential for benefit and highlight the relative safety of antidepressants. In contrast, in the face of uncertainty, we maintain that ‘less is more’ in management of the frail elderly.

## Data Availability

We found nine DBRCT of antidepressants in older adults. We were able to conduct meta-analysis, as the included trials provided data for outcomes in treatment and placebo groups. The data from the included studies was published in peer-reviewed manuscripts, which are available on PubMED/MEDLINE and/or upon request from the study authors.

## Abbreviations

CADTH: Canadian Agency for Drugs and Technologies in Health
CGI-I: Clinical Global Impression-Improvement
DBRCTs: Double-blind, randomized, controlled trials
DSM: Diagnostic and Statistical Manual of Mental Disorders
HDRS: Hamilton Depression Rating Scale
ITT: Intention-to-treat analysis
MADRS: Montgomery-Åsberg Depression Rating Scale
MOOD-HF: Mortality, Morbidity, and Mood in Depressed Heart Failure Patients
NaSSAs: Noradrenergic and specific serotonergic antidepressants
NNH: Number Needed to Harm
NNT: Number Needed to Treat
NORA: Nordic Research on Ageing
PRISMA: Preferred Reporting Items for Systematic reviews and Meta-Analyses
RR: Relative risk
SADHART-CHF: Sertraline Against Depression and Heart Disease in Chronic Heart Failure
SNRI: Serotonin-norepinephrine reuptake inhibitors
SSRI: Selective serotonin reuptake inhibitor
